# Parasitic leiomyoma of the greater omentum: a case report and literature review

**DOI:** 10.1093/jscr/rjad733

**Published:** 2024-01-30

**Authors:** Zhiman Huang, Yongzhou Wang, Hongyan Lei

**Affiliations:** Department of Gynecology, The Affiliated Traditional Chinese Medicine Hospital of Southwest Medical University, Luzhou 646000, Sichuan, China; Department of Gynecology, The Affiliated Traditional Chinese Medicine Hospital of Southwest Medical University, Luzhou 646000, Sichuan, China; Department of Gynecology, The Affiliated Traditional Chinese Medicine Hospital of Southwest Medical University, Luzhou 646000, Sichuan, China

**Keywords:** parasitic leiomyoma, the greater omentum, fibroid

## Abstract

Parasitic leiomyomas (PL), also known as free leiomyomas, which occur outside the uterus and rarely happen in clinical practice. They are usually reported in women of reproductive age who underwent hysterectomy or myomectomy and frequently present with symptoms such as abdominal pain and distention. In fact, it is hard to determine the nature of the mass according to the imaging examination and clinical manifestation. At present, the most common treatment involves an abdominal or laparoscopic surgery in order to remove the mass and perform the next step of treatment based on the histological diagnosis. In this case report, we describe a 35-year-old woman with a 12.4 × 9.3 × 9.8 cm^3^ PL with blood supply from the greater omentum. Considering the prolonged menstruation of the patient, she underwent the hysteroscopic and laparotomy exploration. The mass was confirmed as smooth leiomyoma with necrosis by the immunohistochemical examination. The patient had a good recovery and being discharged seven days after the surgery. The patient is still in the follow-up.

## Introduction

It is well known that fibroids also called uterine leiomyomas (UL) are the most common benign tumor in the female genitalia which consists of smooth muscle and connective tissue. They may occur in in the reproductive age group, with an estimated prevalence ranging from 4.5 to 68.6% while the majority are asymptomatic [1]. However, it’s unusual happened outside the uterus, such as the omentum, mesentery, peritoneum and organs of abdominopelvic cavity. The actual reason of parasitic leiomyomas (PL) is unknown so far, though there are several widely recognized hypotheses. Parasite leiomyomas were originally brought up by Kelly and Colon in 1909; they were thought to derived from subserosal fibroma that twisted off their uterine vascular pedicle and survived via neovascularization of adjacent organs [2]. According to the present literatures, the most convinced reason may be that the PL is a rare complication caused by the residual fragments after fibroids’ removal [3]. The actual clinical prevalence of parasitic fibroids is presumed to be higher due to the non-specific nature of early clinical symptoms and the limitations of case reports. Here we present a young women with PL manifested as abdominal pain and distention with two previous cesarean deliveries and one laparoscopic myomectomy.

## Case report

### Clinical findings

**Figure 1:**
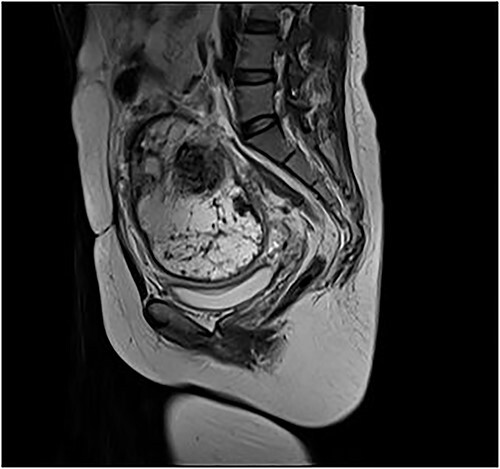
Pelvic enhancement MRI showing large intra-abdominal mass.

A 35-year-old female was diagnosed with prolonged menstruation for more than 10 years and a 2-day history of lower abdominal distension and distention to our gynaecology department. She is a reproductive woman, with two previous cesarean sections, 11 and 4 years ago, respectively, and one laparoscopic myomectomy 9 years ago. Furthermore, there were no reports of abnormalities after the operations in the follow-up. On examination, she appeared mildly anxious and pessimistic due to the large mass but with normal vital signs. A mass of about 12 × 9 cm^2^ with moderate texture and mobility was obtained in the right side of the pelvis by palpation. Her hemoglobin was 117 g/L, red blood cell count was 3.88 × 10^9^ g/L, white blood cell count was 16.17 × 10^9^/L, platelet count was 256 × 10^9^/L and the percentage of neutrophils was 87.5%.Additionally, her CA-125 was 56.04 U/ml and HE-4 was 53.70 pmol/L. Gynecologic transvaginal ultrasound revealed the following: (i) weakly echogenic nodules in the parenchyma of the uterus: myoma? (ii) No echo at the lower incision of the anterior uterine wall: diverticulum? (3) Mixed echogenic mass in the pelvis: teratoma? (iv) Mixed occupancy in the left adnexal area: tubal effusion? Pelvic enhancement MRI revealed the following (Fig. 1) (i) Right pelvic adnexal area showing mass-like abnormal signal: chocolate cyst? Encapsulated cystic lesion? Teratoma? (ii) Possible cyst in the left adnexal region; the isthmus of the anterior wall of the uterus is symptomatic. (iii) Multiple irregular nodular abnormal signals in the anterior and posterior margins of the lower uterine segment: myxoma with degeneration? Other? (iv) Multiple small lymph nodes in the pelvic wall bilaterally; small amount of pelvic fluid; soft tissue swelling around the pelvic wall. Considering that the large mass in the pelvic and more than 10 years of prolonged periods of the patient, she underwent the hysteroscopic and laparotomy exploration after the control of infection.

### Surgery findings

Hysteroscopic findings: normal size cervix and smooth cervical canal, about 0.5 × 0.3 cm^2^ depressed weak area from the endocervix to the lower part of the anterior wall of the uterine cavity, covered with a thin layer of light red endometrium in the surface, uterine cavity depth of about 8 cm, endometrial thickness of about 0.7 cm, uterine cavity was disorganized, full of finger-like protruding superfluous organisms with tips, red in color and soft in texture, maximum diameter of about 1.0 cm, bilateral fallopian tube openings clearly visible.

### Laparotomy findings

**Figure 2:**
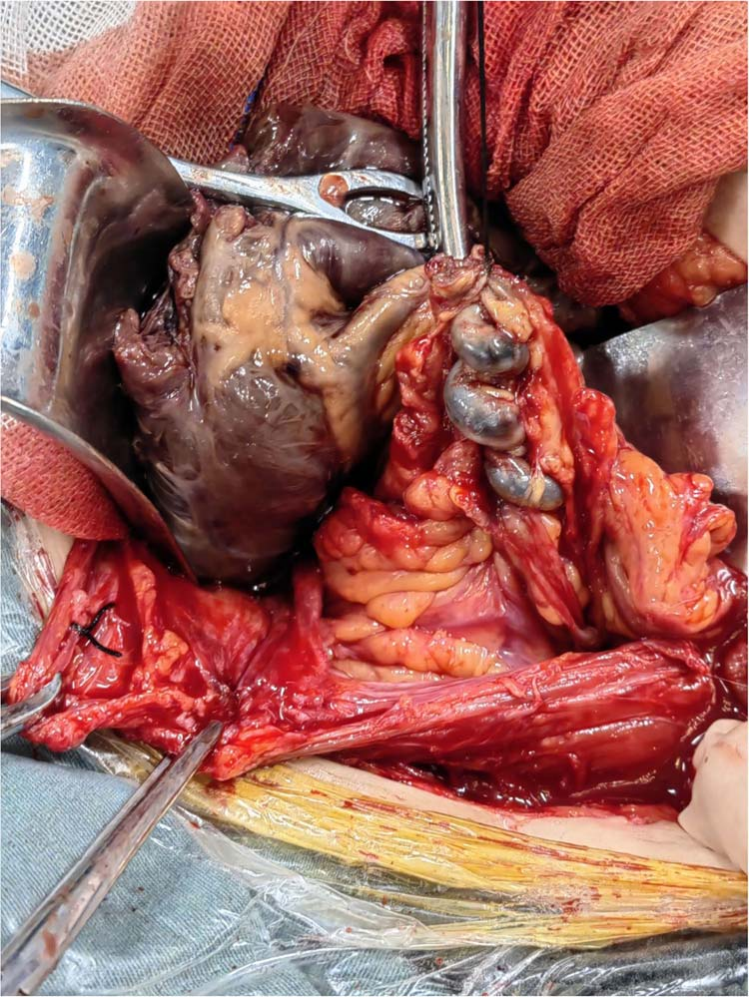
Resected parasitic leiomyoma along with its vascular pedicle.

A large mass (12 cm × 10 cm × 10 cm), isolated from the uterus but connected to the greater omentum, was seen with a purplish-brown appearance and an intact envelope (Fig. 2). The left posterior wall of the mass was adherent to the greater omentum, where the greater omentum was twisted, and a about 5 cm-long twisted thickened vessel with a diameter of about 0.3 cm was seen in the greater omentum, with a bluish-purple appearance. The uterus was normal in size with intact envelope, and multiple hard nodules were seen in the plasma layer of the lower posterior uterine wall, with a maximum diameter of about 0.2 cm. There were no exact abnormalities in the appearance of all segments of the intestinal canal, and the appendix was normal in size with smooth surface. Dissection of the isolated abdominal mass revealed the following: the mass showed cystic solid changes and the solid component was predominant with a dark red appearance. The texture was rotten, which seemed to be fibrotic necrosis. And the cystic fluid was about 300 ml, brown in color and the texture was clear. The intraoperative cryopreservation tended to be benign lesion.

### Pathology findings

(i) ‘Intrauterine tissues’: endometrial polyps; (2) ‘nodules of posterior wall of the uterus’: leiomyoma; (3) ‘mass of the pelvic’: leiomyoma with necrosis. On immunohistochemical studies of the large mass in the pelvic, it was found to display lesional cells which were positive for smooth muscle actin, desmin and KI-67(<1%). The tissue was also negative for Inhibin-α, S100, PAX8, EMA, STAT-6 and CR. These findings are characteristic of a leiomyoma. The patient was anxious before the intervention; postoperatively, she was happy and satisfied about the outcome. On postoperative day 8, she was discharged home. And now the patient is still in the follow-up.

## Discussion

UL are idiopathic benign fibromuscular tumors in women characterized by the presence of the female genitalia. The International Federation of Gynecology and Obstetrics made a secondary, and tertiary subclassification system for leiomyoma according to their number, size and location, and PL is involved in the category 8, with all the extrauterine leiomyomas and unusual extrauterine variants of leiomyomas [4]. Parasitic fibroma has no connection with the uterus and obtains its blood supplies from the nearby organs and tissues.

### Etiology

To date, there are several possible presuppositions including iatrogenic seeding, the influence of female sex hormones (estrogen and progesterone) and genetic components. With the prevalence of parasite myomas within the last 20 years, the most plausible hypothesis is that during the hysterectomy or myomectomy especially the morcellation, the fragments of the uterus and myoma can be implanted into the abdominopelvic iatrogenically [5]. And the overall incidence of parasitic myomas after laparoscopic morcellation was 0.12–0.95% [6]. Power morcellation, also called electromechanical morcellation, is an instrumental method intended to shred and easily remove organs and tissues through a laparoscopic access port, and it often carried out in gynecology operations, such as laparoscopic myomectomy [7]. Patients who otherwise would not be eligible for minimally invasive surgery (i.e. those with a large uterus or myomas) could benefit from laparoscopic advantages [8]. Accordingly, in 2014, the Food and Drug Administration announced negative statement about the morcellation use due to the risk of potential spreading of cancerous tumor cells, notably uterine sarcomas [9]. However PL occurring in patients who had no prior surgeries cannot be explained by this etiology. In the case presented by Khan *et al.*, a 45-year-old woman with no prior surgeries complaining increasing abdominal girth over past several years, presented as diffuse abdominal pain and obstipation accompanied by bouts of vomiting concerning for a small bowel obstruction and was found to have a 12 × 12 × 8 cm^3^ parasitic leiomyoma of the greater omentum with a small fibrotic attachment to the uterus [10]. This kind of PL possibly originates from previous subserosal pedunculated leiomyomas that lost their attachment to the uterus due to torsion around their peduncle and attempt to access the omental vessels to restore their vascularization [11]. Sex hormones fluctuate significantly during different special periods in women, among which estrogen and progesterone represent the greatest changes. Studies have shown that estrogen can stimulate subcutaneous mesenchymal cells to proliferate and differentiate into myoblasts, fibroblasts, myofibroblasts and even deciduous cells, and can also induce progesterone receptor expression and promote activation [12]. Progestogens may promote cell proliferation, blood vessel formation and maintenance of blood supply to tumors by activating epidermal growth factor, which stimulate fibroid growth, and the combined effect of estrogen and progestin is more active than estrogen alone [13]. Takeda *et al.* presented one pregnant patient with a history of laparoscopic myomectomy 2 years prior, who received conservative observation during this period and there was no significant change of the mass; Preconception ultrasound suggests a pelvic mass about 1.4 cm in diameter, but the tumor grows rapidly during pregnancy, reaching 7 cm in diameter at 5 weeks’ gestation [14]. This suggests that fibroids will grow during pregnancy, which may be related to higher hormone levels in the patient or increased sensitivity to hormones. So the PL is supposed to grow slowly and even may shrink after menopause; however, there are several cases of the postmenopausal women with a large PL. Brakeleer *et al.* reported a postmenopausal woman without previous uterine surgery diagnosed as abdominal swelling and back pain, who was found with a gigantic parasite myoma of 19 kg attached to the retroperitoneal [15].

### Clinical diagnosis

It is hard to diagnose PL just by imaging examination, clinical manifestations and medical history of the patient in the early stage, which often causes a diagnostic dilemma. There is no literature reported on the specific serum markers of PL, and it is easy to be misdiagnosed as teratoma, chocolate cyst, gastrointestinal stromal tumor, lymph node sarcoidosis, etc. The symptoms of the PL patients vary from the location, number and size of the mass. It is estimated that the majority (93%) of PL occur in pelvis presented as abdominal pain and distention [16]. The early clinical manifestations of patients are non-specific, and compression symptoms may occur when the mass gradually increases in the later stage, such as ureteral hydronephrosis, frequent micturition, constipation, incomplete intestinal obstruction and even intestinal perforation. Laibangyang *et al.* reported a 63-year-old woman with a surgical history significant for two cesarean sections and an abdominal myomectomy 20 years prior, with symptoms concerning for a small bowel perforation, who underwent an emergent exploratory laparotomy, verifying it was an abdominal leiomyoma with ischemic necrosis and hemorrhage measuring 42 × 30 × 13 cm^3^ that caused multiple sites perforation containing pus and feculent material from the perforated site finally [5]. Except the compression symptoms, some PL were detected during other surgical procedures accidentally, Varun *et al.* reported a pregnant woman who was found with PL during an emergency caesarean section for fetal head descent [17]. Decleer *et al.* reported a 65-year-old patient in whom a large tumor mass was detected during a routine gynaecological examination [18].

### Treatments and preventions

Though most of PL are benign tumors, unexpected lesions are possible, such as leiomyosarcomas. Leiomyomas may contain small areas with malignant transformation that escape initial diagnosis but later can give rise to local recurrences and metastases [19]. Besides, some PL grows into a large mass causing the abnormality of organs’ function or acute abdomen [5, 15]. And now the mainstay of treatment for PL is complete surgical resection via laparoscopy or laparotomy [20]. What’s important is that any attachments to the uterus should be noticed, given a variant of PL in patients without prior surgery and meticulous examination of mass’ parasitic blood supply is crucial to safe resection and good outcomes for patients [10]. Due to the ‘ iatrogenic seeding ’ theory, a careful inspection and thorough washing of abdominopelvic cavity should be done after the procedure [21]. Regular follow-up of patients with uterine fibroids and regular gynecological examinations of women are extremely significant for the prevention of parasitic fibroids, relative acute abdomen and parasitic fibroid lesions.

## Conclusion

PL is a rare subtype of uterine leiomyomas presented with vague symptoms. They are mostly found incidentally or due to pressure symptoms. Diagnosis by imaging examination is challenging and treatment consists of surgical resection. Though there are possible presumptions, the actual etiology of parasitic fibroids is still unclear. This unique case, a large parasitic leiomyoma with necrosis in a reproductive woman with previous uterine surgery, suggests taking this diagnosis into account in future clinical cases presenting with large abdominal masses.
